# The Effect of Detoxification on Sleep: How Does Sleep Quality Change during Qualified Detoxification Treatment?

**DOI:** 10.1155/2018/9492453

**Published:** 2018-12-20

**Authors:** Peter Neu, Yvonne Sofin, Heidi Danker-Hopfe

**Affiliations:** ^1^Jewish Hospital Berlin, Clinic for Psychiatry and Psychotherapy, Heinz-Galinski-Str. 1, 13347 Berlin, Germany; ^2^Charité, Universitätsmedizin Berlin, Corporate member of Freie Universität Berlin, Humboldt-Universität zu Berlin, and Berlin Institute of Health, Department of Psychiatry, Hindenburgdamm 30, 12203 Berlin, Germany; ^3^Charité, Universitätsmedizin Berlin, Corporate member of Freie Universität Berlin, Humboldt-Universität zu Berlin, and Berlin Institute of Health, Competence Center for Sleep Medicine, Campus Benjamin Franklin, Hindenburgdamm 30, 12203 Berlin, Germany

## Abstract

*Aims. *Sleep disturbances are common in addiction and withdrawal. This study examined the course of sleep quality in a population of alcohol dependent patients during qualified detoxification treatment in a psychiatric hospital.* Methods. *The Pittsburgh Sleep Quality Index (PSQI) was administered to 77 electively admitted alcohol dependent patients hospitalized for qualified detoxification treatment. Sleep quality was measured at admission and at discharge.* Results. *The prevalence of bad sleep as measured by a PSQI-score > 5 was 70.1% at admission. During detoxification, male and female patients were equally affected by sleep disturbances and improvement of sleep was not significantly different between males and females. The PSQI score at admission predicted the change of the PSQI score during qualified detoxification treatment. After inpatient detoxification, sleep disturbances persisted in 59.7% of the patients.* Conclusions. *Contrary to our expectations, the average patient's sleep quality improved in our study after two weeks of detoxification treatment. Sleep disturbances nevertheless persisted in almost two-thirds of the patients. In the view of that finding, patients may require individual evaluation of sleep quality and insomnia-specific treatment in the course of detoxification therapy.

## 1. Introduction

The aetiology of sleep disturbances in addiction seems bidirectional. Research has shown that sleep disturbances experienced early in life predispose an individual to develop a substance use disorder [1] while in turn substance abuse contributes to sleep problems [2]. Repeated administration of substances of abuse can alter the homeostatic balance of neurotransmitters such as acetylcholine [3], dopamine, glutamate [4, 5], GABA [6], norepinephrine [7], and hypocretin/orexin [8] which leads to tolerance and contributes to the development of dependence. Many of the same neurotransmitter systems that are affected by substances of dependence are involved in the regulation of circadian rhythms and sleep physiology [9]. As a result, sleep disturbances are common among people with substance use disorders [10]. Insomnia is highly prevalent in patients with addiction during active use of the substance [11]. Substance use can exacerbate sleep difficulties, which in turn present a risk factor for substance use or relapse [12]. Sleep problems can hereby occur during withdrawal, but have also been found to persist long after withdrawing from the drugs [13]. Nearly 70 % of the patients were admitted for detoxification report sleep problems prior to admission [14]. Alcohol is widely used as a sleep-promoting agent in self-medication of insomnia complaints [15]. Thus it is not surprising that sleep problems are extremely common in early alcohol recovery [16]. Individuals with alcohol dependence frequently report poor quality of sleep [17, 18]. Intoxication with alcohol produces prolonged latency to sleep, decreased total sleep time, and decreased percentage of REM (rapid eye movement) sleep [2]. Prolonged abstinence from alcohol leads to sleep fragmentation, a higher level of arousal during sleep, and an increase in the amount of REM sleep [19, 20].

The aim of the present study was to determine the frequency of bad sleep and the development of sleep quality in a population of alcohol dependent patients hospitalized for qualified detoxification treatment. We hypothesized that 1. sleep quality deteriorates after alcohol detoxification and that 2. bad sleep quality might serve as a predictor for relapse and subsequent unplanned treatment drop-out.

## 2. Methods

### 2.1. Setting and Treatment Procedure

This study was approved by the Ethics Commission of the Charité, Universitätsmedizin Berlin (application number EA1/250/12), and was conducted on two specialized inpatient units for qualified detoxification treatment of addiction diseases in a psychiatric hospital in Berlin, Germany. Actively used, treatment-seeking alcohol-dependent patients were recruited for participation. All fulfilled the DSM-IV criteria for substance addiction and gave their written informed consent to participate in this study. Exclusion criteria were dependent of substances other than alcohol and noncapacity of giving informed consent. The patients were admitted electively for qualified detoxification treatment except for emergency admissions. We applied the qualified detoxification treatment following the methods of Sofin et al. 2017 [21]. During detoxification from alcohol, withdrawal symptoms had to be treated with tapered doses of clomethiazole in the first 2 to 3 days of hospitalisation in 38 patients. Instead of benzodiazepines, clomethiazole with a short elimination half-life of 3 hours was administered to treat symptoms of acute alcohol withdrawal as benzodiazepines are characterised by an elimination half-life of several hours to days; thus the administration would have distorted our results.

### 2.2. Measures of Sleep Quality

Sleep quality was assessed by the Pittsburgh Sleep Quality Index (PSQI), a self-administered instrument to assess sleep duration, disturbance, latency, efficiency, quality, daytime sleepiness, and medication use [22]. The questionnaire consists of 18 items which are assigned to 7 components, each of which can assume a value range of 0 to 3. The total score results from the summation of these component scores can vary from 0 to 21, whereby a higher degree corresponds to a reduced sleep quality. In the year 2012 it was administered twice to 77 alcohol dependent patients. The first PSQI was filled out earliest on the fourth day of hospitalisation after the physical withdrawal from alcohol was completed and clomethiazole was discontinued. With this first questionnaire, the patient's sleep quality of the 4 weeks prior to the hospitalisation was captured. To asses sleep quality during inpatient detoxification treatment after alcohol and, where applicable, clomethiazole were withdrawn; the PSQI was filled out a second time immediately prior discharge. The average treatment took between 12 and 16 days, but could last longer in case of persisting withdrawal symptoms or particularly severe general condition.

Patients who failed to give complete responses to all items were excluded from analysis, as well as all patients that dropped out from treatment and refused to fill out the second questionnaire before leaving the hospital. A PSQI score of greater than five was used to indicate bad sleep quality.

### 2.3. Data Analysis

Statistical analyses were carried out using SAS Version 9.4 (Statistical Analysis System) software by the SAS Institute. Changes between PSQI scores from admission to discharge were calculated using a t-test for paired observations. For nominal and ordinal scaled variables, the analysis was carried out with the log likelihood qui square test. For interval scaled data, the relationship between the respective variable and premature discharge was analysed with a t-test for independent samples (if normal distribution was assumed) or with a Wilcoxon 2-sample test (if normal distribution could not be assumed). Normal distribution was tested using the Kolmogorov-Smirnov test with a two-sided significance level of p < 0.01. Tests on group differences were examined with a two-sided significance level of p < 0.05. General Linear Models were used to assess the predicting power of age, duration of dependency, and the dichotomous variable patient sex on sleep quality at admission and at discharge. For the individual changes of sleep quality from admission to discharge, the PSQI at admission and whether patients dropped out from treatment or not were additionally considered as independent variables.

## 3. Results

### 3.1. Sample Characteristics

The total number of patients who participated in this study was 77 of whom 76.6% (59) were males. The mean age of the patients was 47.7 (±12.2) years with a range from 20 to 73 years. Males and females did not differ significantly in age (males: 48.4 ± 11. 4 years; females 45.4 ± 14.8 years, p = 0.4162). Half of the sample (52.0%) suffered from addiction for more than 15 years. The mean addiction duration was 16.6 (± 10.8) years. On average males suffered significantly longer (p = 0.0458) from addiction than females (males: 18.0 ± 10.9 years; females: 12.3 ± 9.2 years). 38 (49.4%) patients had to be treated with clomethiazole at tapered dosage for up to 3 days. Another sedative medication was not applied.

### 3.2. Sleep Quality of the Sample

**Figure 1:**
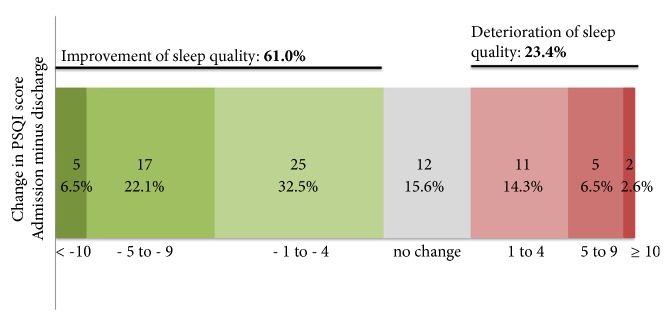
Change of PSQI score during detoxification treatment.

At admission, a total of 54 (70.1%) of the 77 patients had bad sleep quality as indicated by a PSQI score greater than five. At discharge, the quantity of patients with sleep disturbance (PSQI > 5) fell to 59.7% (N = 46). Sleep quality of the entire sample thus improved significantly by 2.0 ± 4.8 score units (p = 0.0006). Mean PSQI at admission measured 9.1 (± 4.6) and fell to 7.1 (± 3.6) at discharge and hence improved by 22.0%. Improvement of sleep was not significantly different for male and female patients (p = 0.7306). We found no significant differences for the PSQI score at admission for men (9.3 ± 4.82) and women (8.6 ± 3.8, p = 0.6552) and for the PSQI score at discharge (women: 6.7 ± 3.9; men: 7.3 ± 3.52, p = 0.5780). The proportion of bad sleepers (those with a PSQI score > 5) was not statistically significant between men and women, neither at admission (p = 0.8238) nor at discharge (p = 0.6802). During detoxification, sleep quality improved in 61.0% of the patients which is reflected in decreases of the PSQI score up to 12 units of score. In 23.4% sleep quality deteriorated as reflected by an increase of the PSQI score up to 11 units (Figure 1). The PSQI did not change during detoxification treatment in 15.6% of the patients.

With regard to the classification of the patients into good or poor sleepers, 68.8% did not change their classification. 19.5% of the patients remained good sleepers while 49.4% persisted to be poor sleepers. Nevertheless, 20.8% of the patients converted from poor to good sleepers, while 10.4% of the good sleepers became poor sleepers during detoxification treatment.

### 3.3. Univariate Analysis of the Change of Sleep Quality during Detoxification Treatment

To further assess the change in sleep quality during qualified detoxification treatment, the PSQI on admission and at discharge as well as individual changes were analysed with regard to patient sex, treatment outcome (regular termination versus unplanned treatment drop-out), and duration of dependency (≤ 15 years or > 15 years). The results of the univariate analysis are summarized in Table 1. There were no significant differences in sleep quality as assessed by the PSQI between male and female patients, neither at admission, nor at discharge nor at the level of individual changes. Effect sizes were negligible.

In this study, five patients (6.5%) prematurely dropped out of treatment. On average, patients that dropped out had higher PSQI values at admission as well as at discharge compared to the patients that completed detoxification treatment successfully. Although not significantly different, the patients with completed treatment had improved sleep by 1.1 score units more than the patients that prematurely dropped out of treatment. Effect size for differences at admission and discharge was small. Duration of dependency did not affect sleep quality at admission and at discharge nor did the duration of dependency affect the change during detoxification. Again, the effect sizes were small (< 0.50).

### 3.4. Regression Analysis on the Influence of Age on Sleep Quality during Detoxification

**Figure 2:**
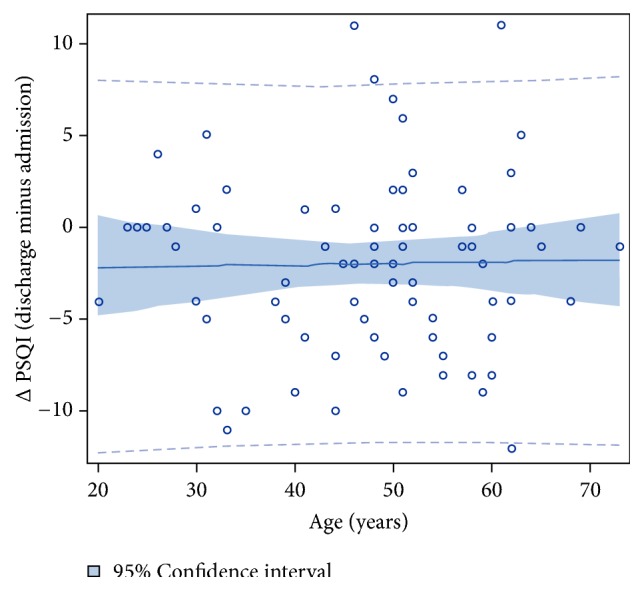
**Plot of changes in the PSQI score from admission to discharge by age.** Regression: ∆PSQI= - 2.3 + 0.007*∗*age (p = 0.8785; R^2^ = 0.0003).

In addition to the effect of sex, treatment outcome and duration of dependency on sleep quality during qualified detoxification treatment, we further analysed a possible influence of age on sleep quality during detoxification treatment. Regression analysis showed that the mean PSQI at discharge was about 2 points lower than PSQI at admission irrespective from the patient's age (p = 0.7101; Figure 2).

### 3.5. Regression Analysis of the Change in PSQI in relation to PSQI at Admission

**Figure 3:**
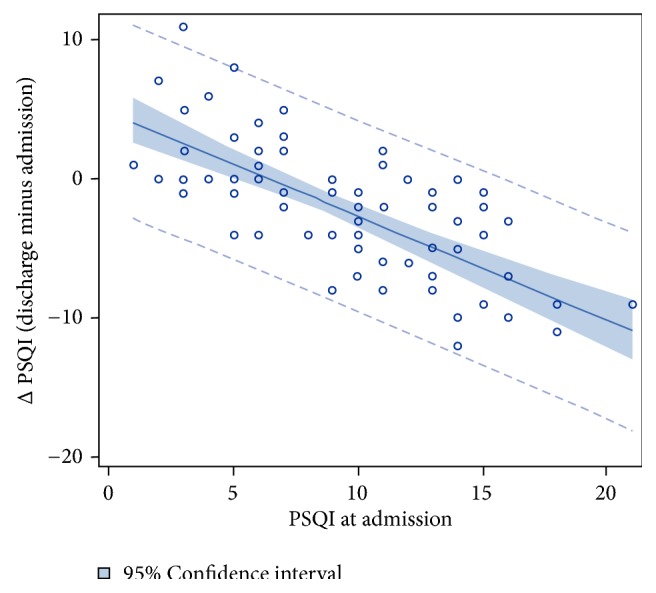
**Regression analysis of the change in PSQI in relation to PSQI at admission.** Regression: ∆PSQI= 4.9- 0.752*∗*PSQI at admission (p < 0.0001; R2 = 0.5096).

Figure 3 shows the regression analysis of the PSQI change after detoxification treatment in relation to the PSQI at admission. Patients with low or no sleep disturbances at admission did not or only slightly improve their sleep. Whereas patients with bad sleep on admission, indicated by higher PSQI scores, improved sleep quality significantly (p < 0.0001), patients with a better sleep at admission did not improve their sleep as much as patients with pronounced bad sleep quality during detoxification treatment. The worse the sleep quality at admission, the more considerably was the revealed improvement. Overall sleep quality at admission explained 51.0% of the change in the PSQI score from admission to discharge.

### 3.6. General Linear Model for Prediction of PSQI at Admission, Discharge, and PSQI Change during Treatment

Finally, a general model with age, sex, and duration of dependency was calculated to identify correlates of sleep quality at admission and at discharge as assessed by the PSQI score (Table 2). Analyses revealed that none of these factors predicted PSQI at admission significantly (all partial *η*2< 0.06). Overall the model was not statistically significant (p = 0.2234) and predicted less than 6% of the variation of the PSQI score at admission. For the PSQI score at discharge the variables age, sex, and duration of dependency were again not statistically significant (p = 0.4216). For the change in PSQI from admission to discharge two additional factors were included in the analysis: the PSQI at admission and whether the patient dropped out from treatment or not. The model explained 51.9% of the change in the PSQI score, with the PSQI at admission (p < 0.0001, *η*2 = 0.514) being a significant predictor.

## 4. Discussion

The aim of the present study was to examine sleep quality after physical withdrawal from alcohol during inpatient qualified detoxification treatment in alcohol dependent patients. The main findings of our study were as follows. (1) Male and female patients were equally affected by sleep disturbances and improvement of sleep was not affected by the patient's sex. (2) Sleep quality was independent of age and duration of dependency. (3) During detoxification, sleep quality slightly improved in 61% of the patients. (4) Nevertheless, 68.8% did not change their classification as good respectively poor sleepers. (5) When discharged, the PSQI score of patients who prematurely dropped out of treatment did not significantly differ from the PSQI score of patients who completed detoxification treatment successfully. Thus, the PSQI score was not a predictor for unplanned treatment drop-out. (6) PSQI score at admission was a significant predictor of the change of sleep quality during detoxification treatment.

70% of our patients reported sleep disturbances at admission indicated by a PSQI score greater than five. This finding meets our expectations as corresponding numbers were reported in literature. Roncero at al. [14] found insomnia in 71% of the patients admitted for alcohol detoxification whereas Escobar-Córdoba et al. [23] even reported rates of 89% (men) and 100% (woman) of patients with PSQI scores >5, respectively. There were no differences in sleep quality between male and female patients. Data about the effect of the patient's sex on sleep quality during detoxification is inconsistent. Past research of Kolla et al. [16] is in accordance with our study whereas others [24] found that insomnia during early alcohol recovery was predicted by female gender. In the general population in turn, insomnia symptoms are more frequently in women than men [25, 26]. Our results are limited by the fact that only 23% of our patients were female. Further investigation should be carried out to verify the influence of the patient's sex on sleep quality during alcohol detoxification.

In the present work, 60% of the patients still had sleep disturbance at discharge; however, sleep quality improved in 61% of the patients. Although sleep problems have been found to persist long after withdrawal [13], patients might have benefitted from relearning to maintain a regular daily structure during their hospitalization, which often got lost during years of addiction. In nearly one-quarter of our patients a deterioration of sleep was observed which again can be explained by the negative impact of withdrawal on sleep. Additionally, it can be concluded that sleep might have been disrupted by room neighbours and the unfamiliar environment in hospital, which both might have contributed to a decrease of sleep quality. It does not surprise that almost 68.8% of our patients did not change their classification as the mean decrease of the PSQI score was only 2 score points at the individual level. Moreover, detoxification happens in an early stage in the long process of recovery. As sleep problems maintain in the first weeks of abstinence, it can be assumed that a further improvement could have been observed over time, as the average duration of detoxification treatment of two weeks was not long enough to improve sleep. As sleep disturbance constitutes a major risk factor for relapse [27], inpatient detoxification treatment might be enriched with programs teaching knowledge how to improve sleep without the use of medication to strengthen patients to revise sleep habits and correct misconceptions about sleep and insomnia. Interestingly, in our study, the reduction of PSQI score after detoxification did not differ significantly between patients that completed treatment regularly and patients that dropped out prematurely from treatment. Descriptive statistics indicate that improvement in sleep is approximately 0.9 score units higher in treatment drop-outs (-2.8) than in those patients who regularly completed the detoxification program (-1.9). However, this difference is not statistically significant. This might be due to the small sample size of patients who dropped out and for which PSQI data are available. The drop-out rate in this study was remarkably low (6.5%) compared to previous studies reporting drop-out rates of 30% [28, 29]. This discrepancy might be explained by the fact that study participation was voluntary and that mainly highly motivated patients participated in this study. It can be assumed that patients with high risk of unplanned drop-out, e.g., due to missing therapy motivation or decreased frustration tolerance, were not willing to participate in our study at all. Furthermore, patients that missed to fill out the PSQI at discharge due to treatment drop-out were excluded from data evaluation. Only data from patients that completed both questionnaires were taken into consideration. Nevertheless, patients that dropped out of treatment had poorer PSQI values compared to patients that completed detoxification treatment. It is conceivable that the improvement of sleep quality during withdrawing did not fulfil the patient's expectations and weakened their therapy motivation.

In our study, duration of dependency and age did not predict sleep quality during detoxification. These results suggest that alcohol has an effect on sleep disturbances independent of duration of use and age. It can be assumed that age-related factors like life experience and profound knowledge of the patient's addiction disease do not enable patients to influence or tolerate impaired sleep quality. Nevertheless, substance use disorders and in consequence sleep disorders can be influenced by the stage of development [2] but could not be shown in our study as only one out of 77 patients was younger than 21 years. PSQI score at admission was found to predict the PSQI score change during detoxification. Our findings might be an indication that the PSQI at admission might serve as indicator for addiction severity and in turn as indicator for the extent of the overall influence of the imminent detoxification on sleep. It is plausible that patients with a low PSQI score at admission do not improve to the same extent as patients with severely impaired sleep physiology.

This study has some limitations. Sleep quality was only observed at the beginning and the end of the detoxification treatment but not beyond. Additional long-term observations in the weeks after inpatient treatment shall complete the picture of sleep disturbances in addiction treatment. Furthermore, all assessments of sleep quality evaluated in this study were based on patient self-report. The present study does not allow corroboration of the patient's statements made in the questionnaire. In view of the above, additional research enriched with polysomnographic studies supporting the patient's subjective statements made in the PSQI and studies to include the collection of the patient's withdrawal state (e.g., the severity of alcohol withdrawal symptoms according to the CIWA withdrawal score [30]) shall be conducted.

Finally, information about medical conditions or medications that might contribute to insomnia was not obtained. However, different from other institutions, no benzodiazepines were administered to the patients during alcohol detoxification. It is unlikely, but conceivable, that the administration of clomethiazole distorted our results, at least for the 38 patients, were alcohol withdrawal symptoms that had to be treated with clomethiazole. However, half-live-time of clomethiazole is short (3 hours), so that this might certainly not have affected the PSQI at discharge. If there was a considerable impact of clomethiazole on sleep quality, one would have not seen an improvement of sleep quality at discharge.

In summary, insomnia was highly prevalent in the patients included in this study, at admission but also upon discharge from hospital. Overall, sleep quality was slightly improved in most patients following two weeks of qualified detoxification treatment but anyhow remained in 59.7% of the patients. In the light of these findings, patients may require individual evaluation of sleep quality and insomnia-specific treatment in the course of detoxification therapy. Further studies addressing this promising issue are mandatory.

## Figures and Tables

**Table 1:** Univariate statistics of PSQI score at admission and discharge.

	mean ± SD	mean ± SD	p	|effect size|^1^
**Sex**	**Female (n = 18)**	**Male (n = 59)**		

PSQI Admission	8.6 ± 3.8	9.3 ± 4.8	0.6552	0.14
PSQI Discharge	6.7 ± 3.9	7.3 ± 3.5	0.5780	0.15
PSQI ∆	-1.9 ± 4.5	-2.0 ± 5.0	0.7306	0.02

**Treatment drop-out**	**Yes (n = 5)**	**No (n = 72)**		

PSQI Admission	11.0 ± 7.0	9.0 ± 4.4	0.4309	0.44
PSQI Discharge	8.2 ± 2.6	7.1 ± 3.6	0.3499	0.32
PSQI ∆	-2.8 ± 6.9	-1.9 ± 4.7	0.7243	0.18

**Duration of dependence**	**≤ 15 (n = 37)**	**> 15 (n = 40)**		

PSQI Admission	9.9 ± 5.4	8.3 ± 3.6	0.1692	0.36
PSQI Discharge	7.4 ± 4.0	6.9 ± 3.2	0.6085	0.41
PSQI ∆	-2.5 ± 5.2	-1.5 ± 4.5	0.4014	0.23

^1  ^Effect size: small effect, 0.2 < d ≤ 0.5; medium, 0.5 < d ≤ 0.8; large, d > 0.8.

**Table 2:** General linear model for prediction of PSQI at admission, discharge, and PSQI change during treatment.

**Factor**	**PSQI at admission**		**PSQI at discharge**		Δ** PSQI (discharge – admission)**	
	p	Partial *η*^2^	p	Partial *η*^2^	p	Partial *η*^2^
Age	0.1401	0.030	0.1813	0.024	0.3453	0.013
Sex	0.3594	0.012	0.4183	0.009	0.5694	0.005
Duration of dependency	0.0446	0.054	0.1296	0.031	0.3221	0.014
Treatment drop-out (yes/no)	N/A	N/A	N/A	N/A	0.6319	0.003
PSQI at admission	N/A	N/A	N/A	N/A	< 0.0001	0.514
Pr> F	F_ (3;73)_ = 1.49 0.2234		F_ (3;73)_ = 0.95 0.4216		F_ (5;71)_ = 15.35 < 0.0001	
R^2^	0.0578		0.0375		0.5194	

N/A = not applicable.

## Data Availability

The underlying data related to our manuscript can be made available by the corresponding author upon request.
